# Comparison of RNA- or LNA-hybrid oligonucleotides in template-switching reactions for high-speed sequencing library preparation

**DOI:** 10.1186/1471-2164-14-665

**Published:** 2013-09-30

**Authors:** Matthias Harbers, Sachi Kato, Michiel de Hoon, Yoshihide Hayashizaki, Piero Carninci, Charles Plessy

**Affiliations:** 1 K.K. DNAFORM, Leading Venture Plaza-2, 75–1, Ono-cho, Tsurumi-ku, Yokohama City, Kanagawa 230-0046, Japan; 2 RIKEN Center for Life Science Technologies, Division of Genomics Technologies, Yokohama, Kanagawa 230-0045, Japan; 3 RIKEN Omics Science Center, Yokohama, Kanagawa 230-0045, Japan; 4 RIKEN Preventive Medicine & Diagnosis Innovation Program, Yokohama, Kanagawa 230-0045, Japan

**Keywords:** CAGE, Template-switching, LNA, Transcriptome, Quantitative sequencing

## Abstract

**Background:**

Analyzing the RNA pool or transcription start sites requires effective means to convert RNA into cDNA libraries for digital expression counting. With current high-speed sequencers, it is necessary to flank the cDNAs with specific adapters. Adding template-switching oligonucleotides to reverse transcription reactions is the most commonly used approach when working with very small quantities of RNA even from single cells.

**Results:**

Here we compared the performance of DNA-RNA, DNA-LNA and DNA oligonucleotides in template-switching during nanoCAGE library preparation. Test libraries from rat muscle and HeLa cell RNA were prepared in technical triplicates and sequenced for comparison of the gene coverage and distribution of the reads within transcripts. The DNA-RNA oligonucleotide showed the highest specificity for capped 5′ ends of mRNA, whereas the DNA-LNA provided similar gene coverage with more reads falling within exons.

**Conclusions:**

While confirming the cap-specific preference of DNA-RNA oligonucleotides in template-switching reactions, our data indicate that DNA-LNA hybrid oligonucleotides could potentially find other applications in random RNA sequencing.

## Background

New generations of high-speed sequencing instruments in combination with dedicated sample preparation protocols can provide results within a few days. We have developed nanoCAGE to identify each transcript by a single sequencing read [1,2]. The nanoCAGE protocol is most suitable for digital expression profiling with benchtop sequencers as it can use a lower number of reads than any RNA-seq method and avoids normalization of read counts over transcript length. Including a cap-enrichment step, nanoCAGE focuses on sequencing tags from the 5′ end of mRNA that directly indicate expression at defined genomic locations, the transcription start sites (TSS), after mapping to a reference genome.

NanoCAGE libraries are prepared in a two-step process comprising a reverse transcription reaction followed by stepwise PCR. During the reverse transcription reaction a template-switching oligonucleotide is added to directly introduce 5′ adapter sequences for PCR and Illumina sequencing. Further, the 5′ adapter can include different barcodes to facilitate multiplex sequencing [3]. The 3′ adapter sequences needed for PCR and Illumina sequencing are introduced by the reverse-transcription primers and the later PCR. We prefer the use of random primers to cover also non-polyadenylated transcripts in the libraries.

Template-switching allows for cDNA preparation from very small RNA amounts, where even protocols have been published for the analysis of single-cells [4-6]. However, the selectivity for the cap-dependent enrichment of 5′ ends in mRNA can vary: template-switching has been used in cap-specific reactions for the preparation of full-length cDNA libraries [7] and 5′ RACE experiments [8], but also unspecific reactions are possible, for example in the preparation of RNA-seq libraries from fragmented RNA [9]. Depending on the intended use of the template-switching reaction, it is therefore very important to find optimal conditions for cap-specific and cap-independent reverse transcription reactions. Commonly RNA-hybrid oligonucleotides are used in template-switching reactions because they provide cap-selectivity [7,10]. The use of DNA oligonucleotides in template-switching reactions has also been reported by [8], but it is hard to draw general conclusions from that work, since, there is no comparison with DNA/RNA hybrids, and the target RNA was prokaryotic, and therefore not capped.

DNA/RNA hybrids have inherent disadvantages compared to DNA oligonucleotides such as their higher price and the intellectual property on their use [10]. We therefore investigated alternatives, with the additional goal of increasing the efficiency or the cap specificity of the reaction. Here we compare nanoCAGE libraries prepared using DNA oligonucleotides as well as RNA- and LNA-hybrid oligonucleotides in the template-switching reactions. LNA (Locked Nucleic Acids) are nucleic acid analogs with a higher DNA and RNA binding affinity than classical DNA oligonucleotides [11]. Therefore we tested whether LNA can mimic RNA-like features in template-switching reactions and increase the performance of nanoCAGE library preparation.

## Results and discussion

Three DNA-LNA hybrid oligonucleotides having 3, 2, or 1 LNA nucleotide at their 3′ end (denoted as L3, L2, L1 respectively) were benchmarked with our standard DNA-RNA hybrid oligonucleotide having 3 RNA nucleotides at its 3′ end (R3), and a DNA oligonucleotide (D3). All oligonucleotides had a GGG-tail at the 3′ end as required for cap-dependent template-switching reactions and different barcodes followed by sequences for PCR and Illumina sequencing. Besides the different barcodes used in multiplex sequencing, all oligonucleotides used in this study had the same sequence. NanoCAGE libraries were prepared in technical triplicates according to our standard protocol [2] rom the same total RNA from rat skeleton muscle. The yields of the first strand cDNA synthesis reactions were measured by qPCR [2], where we found reduced cDNA yields for all reactions compared to the standard R3 template-switching oligonucleotide (Table 1). Therefore we increased the number of semi-suppressive PCR cycles for those nanoCAGE library preparations to obtain the same DNA amounts for all libraries (Table 1). Pooled nanoCAGE libraries were combined for multiplex sequencing on an Illumina MiSeq instrument. Since the MiSeq provides a limited number of reads per library (4,682,200 reads in total, see Additional file 1: Table S1 for details), we assessed the results for the MiSeq run with a reference library made from the same RNA sample but deeply sequenced on an Illumina HiSeq instrument (data not shown). The fraction of reads aligning to promoters was not significantly different (*p* = 0.083, Welch two-sample t-test, also used in all other comparisons unless noted otherwise), indicating that the smaller coverage on a MiSeq is sufficient for nanoCAGE library characterization.

**Table 1:** Yields, amplification, and rates of strand-invasion artifacts in libraries made with the RNA- (R3), DNA- (D3), or LNA-based (L3) TS oligonucleotides

**TS oligonucleotide**	**qPCR yield (Ct value)**	**Semisupressive PCR cycles**	**Strand invasion (%)**
R3	20.4	23	9.1 ± 0.7
D3	23.6	23	73.6 ± 3.4
L1	22.8	23	48.6 ± 6.9
L2	26.2	28	38.6 ± 8.1
L3	27.5	28	35.9 ± 4.2

**Figure 1:**
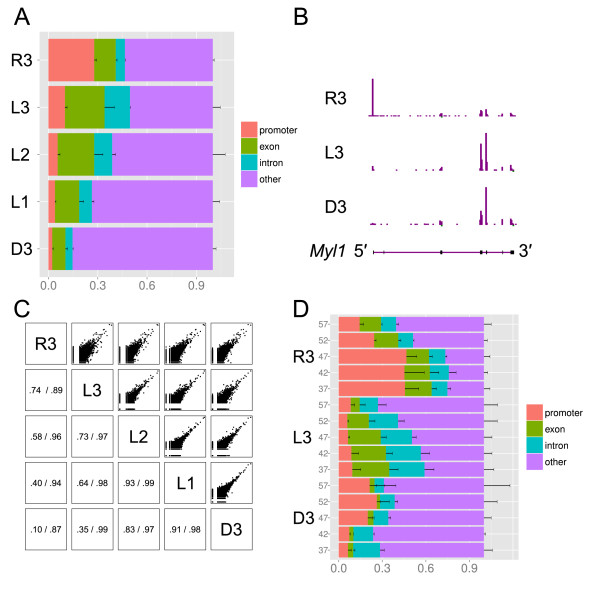
**Genomic features and expression levels of DNA, LNA and RNA-based libraries. (A)** Percentage of reads that align to known genomic features (promoter, intron, exon), for the libraries comparing the template-switching (TS) oligonucleotides R3, L3, L2, L1, and D3. Technical triplicates were averaged and error bars represent standard deviations. **(B)**: Genomic representation of the R3, L3 and D3 libraries on the *Myl1* locus (11 kbp). Aligned CAGE tags are represented as horizontal bars colored in purple for the sense strand and in green for the antisense strand, where the height is proportional to the number of tags in a given genomic bin, after normalizing tag counts per million (TPM) and averaging the triplicates. The highest peaks have the same size in the three tracks, representing 5717, 1665 and 180 TPM values for R3, L3 and D3 respectively. **(C)** Pairwise comparisons between the D3, L1, L2, L3 and R3 libraries. Each square provides data for the pair of libraries defined by the horizontal and vertical intersections with the diagonal. Upper part: Expression plots (logarithmic scale), where each dot represents the normalized number of reads aligning to a reference gene model (same strand only). Technical triplicates were averaged. Lower part: Pearson correlation coefficients before (left) and after (right) removal of strand-invasion artifacts. **(D)** Influence of temperature (indicated by grey labels) on TS with the R3, L3, and D3 TS oligonucleotides. Technical triplicates prepared on random-primed HeLa RNA.

The multiplex MiSeq sequencing run provided sufficient sequencing reads to map [12] between 554,953 and 30,584 CAGE tags to the rat reference genome after discarding reads matching to rRNA sequences or resembling oligonucleotide artifacts (“tag dust”, [13]). We clustered the 5′ ends of the mapped reads into CAGE transcription start sites and counted them into baskets comprising *promoters*, *exons*, *introns*, and *other* regions. The number of reads mapped to *promoters* and *exons* was compared for each library to confirm the cap-specificity of the template-switching reactions (Figure 1A, Additional file 1: Table S1). Here we noted that the L3 oligonucleotide yielded as many mapped reads hitting gene bodies as the R3 oligonucleotide commonly used in template-switching reactions (50% and 47% respectively, *p* = 0.37). Surprisingly, all the nanoCAGE libraries made by D3 and L3 showed higher mapping rates to *exons* than to *promoters* in contrast to the nanoCAGE libraries made by R3 (*promoter* / *exon* = 0.27, 0.44 and 2.13 respectively, *p* < 0.005). This may be explained by a stronger tendency of D3 and L3 to initiate strand invasion, prematurely ending reverse-transcription by hybridizing with complementary regions in the first-strand cDNA before it reaches the end of the mRNA [3] (Table 1). Within individual loci, we found these strand-invasion artifacts on both strands, creating quantities of antisense signal that does not reflect the transcriptome, especially in the D3 libraries (Additional file 1: Table S1). We therefore removed all the clusters that can be explained by strand invasion, using a threshold of two mismatches. Visual inspection of filtered data confirmed the general tendency for lower *promoter*-*exon* ratios in the D3 and L3 libraries (Figure 1B). L1 and L2 libraries displayed an intermediate profile when compared to D3 and L3. Altogether, these observations suggest that only template-switching reactions with RNA-DNA hybrid oligonucleotides allow for cap enrichment.

Next we examined whether all nanoCAGE libraries covered the same set of genes First, we compared the number of different loci detected in each library, after normalizing their sequencing depth to a fixed number of mapped tags by random sampling of 30,000 tags per library. A similar number of genes were detected in each set of technical triplicates (Additional file 1: Table S1), where the coefficient of variation was never higher than 8%. After pooling the triplicates 5,414 (R3), 5,025 (L3) and 5,922 (D3) known loci were identified. Second, we intersected the lists of loci and measured that 2,643 loci were common to all pools. This apparently low overlap is typical in digital expression analysis of samples where the distribution of expression levels is scale-free, because most of the detected loci have low counts, and therefore are likely to be missed even in libraries that are similar by design. To illustrate this, we calculated the number of common loci after replacing the L3 and D3 pools by random samplings of R3 with no replacement, and identified 2,818 common loci. This indicates similar gene coverage by all the libraries despite the low sequencing depth, and also underlines that even with less than 100,000 aligned reads, nanoCAGE libraries can cover thousands different loci. Given the similar gene coverage of the libraries, we then calculated gene-wise Pearson correlation coefficients between the libraries after pooling all the tags aligning to the same gene and obtained correlations ranging between 0.87 and 0.99 (Figure 1C). Lastly, we inspected the significantly over- and under-represented tag clusters in all possible pairwise comparisons of the R3, D3 and L3 triplicates after removal of the strand-invasion artifacts (Additional file 2: Table S2). Using edgeR (Robinson et al., 2010), we found that the R3 libraries had 872 and 626 clusters over-represented compared to L3 and D3 respectively (with false discovery rates lower than 0.05). Respectively, 63 and 69% of the clusters had a known gene symbol and visual inspection confirmed that they corresponded to 5′ regions. The most significantly enriched clusters in R3 compared to L3 were signal recognition particle RNAs (7SL RNA). The same enrichment was also significant, although it did not rank as high, when comparing R3 to D3. Concomitantly, 7SL RNA was also found at significantly higher levels in D3 than in L3. This is the main difference between D3 and L3, where we found only 44 differentially represented clusters in total. Visual inspection of the clusters over-represented in D3 or L3 compared to R3 suggest that they are mostly comprised of remaining strand-invasion artifacts that were below the filter’s threshold, or low-complexity regions that were GT- (D3) or GA-rich (L3), which might reflect the sequence of the barcodes used for these libraries. Altogether, the overlap and differential representation studies confirm that the main differences between the libraries are the prevalence of strand invasion and the distribution of tags within gene models.

To examine whether the specificity of template-switching is dependent on temperature [14], we varied in the next experiment the temperature of the reverse transcription from 37 to 57°C. To further confirm the robustness of our observations across different RNA samples, we prepared these libraries from total HeLa cell RNA. High temperatures of 52 and 57°C caused the formation of large numbers of oligonucleotide artifacts (tag dust) with all types of oligonucleotides (42 ± 11% of all the reads, Additional file 1: Table S1). The annotation of the aligned reads also indicated that lower temperatures at 37°C or 42°C are preferable for RNA and LNA mediated template-switching reactions (Figure 1D). The experiment confirmed with a human RNA source that shifting from RNA to LNA reduces the promoter rate while increasing the coverage of tags mapping within transcripts regardless of the temperature. We explain the higher number of tags aligning within known regions in this experiment compared with the previous one using rat muscle RNA by the better annotation of the human genome in comparison with the rat genome.

## Conclusions

Our results indicate that the cap-specificity of template-switching reactions depends on the nature of the nucleotides interacting with the first-strand cDNA during the template-switching reaction. Interestingly, RNA nucleotides give the highest cap specificity. While DNA/LNA duplexes are also expected to be more stable than DNA/DNA duplexes [15] they showed clearly reduced cap specificity as compared to RNA nucleotides. Therefore, the binding affinity alone does not explain the cap specificity during template-switching reactions. The yield of template-switched cDNAs was also higher with oligonucleotides ending with R3 compared with D3 and L3 (Table 1). We therefore hypothesize that the RNA template-switching oligonucleotides are more efficient with capped templates. A key difference is that capped templates induce the reverse-transcriptase to extend the first-strand cDNA with cytosines because it reverse-transcribes the cap [16-18], while with non-capped templates, extensions are more rare [17], or of a different nature [19,20]. We speculate that only the presence of DNA/RNA base pairs facilitate template-switching at capped ends, and that DNA-DNA, DNA-LNA and DNA-RNA oligonucleotides have the same efficiency on blunt RNA/cDNA hybrids. The higher promoter rate with R3 would therefore reflect an additional production of template-switched cDNAs from capped templates. This dilution of the non-cap-specific signal would also explain the lower proportion of strand invasion in R3.

DNA oligonucleotides have the strongest tendency for strand-invasion, found in almost 75% of the alignments, which makes them impractical for cap selection. A careful assessment of the impact of these strand-invasion artifacts would be necessary before using DNA oligonucleotides for expression profiling. In addition, a large number of the remaining reads did not align in known genes, resulting in a net loss of usable reads for digital expression analysis and for template-switching-based methods in general. Compared to DNA-based libraries, RNA and LNA-based libraries had a deeper coverage over gene bodies, which in the case of LNA comprises mostly non-promoter, exonic signal (Figure 1A). While no longer providing the promoter/TSS specificity of nanoCAGE, LNA-mediated template-switching could become an option in the preparation of digital expression libraries or revised versions of RNA-seq protocols [9]. Further work would be needed to assess whether LNA-mediated template-switching could be preferable for digital expression profiling starting from partially degraded RNA samples, where the cap structure of many transcripts has been lost.

## Methods

All nanoCAGE libraries were prepared in technical triplicates according to [2]. To simplify the library preparation, we used only two different numbers of PCR cycles (Table 1), instead of optimizing for each sample. As a consequence, the number of reads per sample was not optimally balanced. Another possible reason may be that we pooled the barcoded samples (see below and Additional file 1: Table S1) by using 80 ng of an equimassic mixture (not equimolar) of the cDNA PCR products, as templates for the Library PCR (See Salimullah et al., 2011 [2] for details). Nevertheless, this does not affect our analysis since we either normalized the total number of reads, or used methods that are robust to variations of sequencing depth.

The sequences of the template-switching oligonucleotides sequence were as follows, where bases are desoxynucleotides except signaled by a lower case “l” for LNA and “r” for RNA, and where XXXXXX is a sample barcode of sequence CACTGA, GCTCTC or TCGCGT for R3; ATCGTG, CACGAT or GTATAC for D3; ACAGAT, CTGACG or GAGTGA for L1; AGTAGC, GCTGCA or TCGAGC for L2 and ATCATA, CGATGA or TATAGC for L3. The 8 N bases are a unique molecular identifier [21,22], which was not used in this analysis.

>D3

TAGTCGAACTGAAGGTCTCCAGCAXXXXXXNNNNNNNNTATAGGG

>L1

TAGTCGAACTGAAGGTCTCCAGCAXXXXXXNNNNNNNNTATAGG(lG)

>L2

TAGTCGAACTGAAGGTCTCCAGCAXXXXXXNNNNNNNNTATAG(lG)(lG)

>L3

TAGTCGAACTGAAGGTCTCCAGCAXXXXXXNNNNNNNNTATA(lG)(lG)(lG)

>R3

TAGTCGAACTGAAGGTCTCCAGCAXXXXXXNNNNNNNNTATA(rG)(rG)(rG)

Reads were sequenced on a MiSeq instrument (Illumina, USA) version 1 loaded at 10 or 12 pM, using single reads of length 70 or 64 nt, and the standard nanoCAGE sequencing primer [2]. The sequences were analyzed on a Debian system [23] version 6. Barcodes were extracted, linkers were removed, and reads were trimmed to 31 nt with FASTX-toolkit (http://hannonlab.cshl.edu/fastx_toolkit/). Reads resembling empty constructs or oligonucleotide artifacts were removed with TagDust [13]. Reads were then aligned to the rat genome (5.0) and the human genome (hg19) with BWA 0.6.2 [12]. Reads matching the reference ribosomal sequence V01270 (rat) U13369.1 (human) were flagged “failed” with the rRNAdust program, an efficient implementation of Myers’ bit parallel dynamic programming algorithm [24] using both SIMD instructions and threads (written by T. Lassmann). Adding the mitochondrial rRNA (NCBI gene ID 170603) to the filter would cause the removal of approximately one third more sequences. The alignments were post-filtered to remove strand-invasion artifacts as in [3]. The CAGE tags were then clustered following the same principles as in [25], with no expression threshold. The filtering and clustering programs are available in the Additional file 3, with example scripts reproducing the digital expression comparison of the R3, L3 and D3 libraries. The genome annotations were retrieved from ENSEMBL’s biomart server (rat build 70) or the UCSC genome browser (human) and we used custom scripts to extract the coordinates of promoters (which we defined as +/− 100 nt flanking the start site of transcript models). Each CAGE tag cluster was annotated against this reference data using BEDTools [26]. The resulting data was mined and processed using R (http://www.r-project.org/). Subsamplings were done with the ‘vegan’ package and graphs were prepared with the ‘ggplot2’ package and assembled with Inkscape (http://inkscape.org/).

### Availability of supporting data

The FASTQ files are being deposited to DDBJ and are also available at http://genome.gsc.riken.jp/plessy-20130430/NCms10010.fastq.xz (rat muscle) and http://genome.gsc.riken.jp/plessy-20130430/NCms10013.fastq.xz (HeLa). The CAGE tags were then clustered using scripts available at http://genome.gsc.riken.jp/plessy-20130430/PromoterPipeline_20130430.tar.gz and filtered using the rRNAdust program available at http://genome.gsc.riken.jp/plessy-20130430/rRNAdust_1.02.tar.gz and the script available at http://genome.gsc.riken.jp/plessy-20130430/find_strand_invasion-20130307.pl. A digital expression comparison of the R3, L3 and D3 libraries is available at http://genome.gsc.riken.jp/plessy-20130430/analysis.html and its source is available at http://genome.gsc.riken.jp/plessy-20130430/analysis.Rmd.

## Abbreviations

CAGE: Cap analysis gene expression; LNA: Locked nucleic acid.

## Competing interest

CP and PC are co-inventors of the patent 2009/154303 covering the semi-suppressive PCR step in nanoCAGE.

## Authors’ contributions

SK performed the experiments, MdH contributed software, MH and CP analyzed the data, YH and PC provided support, and MH and CP designed the project and wrote the manuscript. All authors read and approved the final manuscript.

## Supplementary Material

Additional file 1: Table S1 Details on the libraries made from Rat muscle and Human HeLa RNA. All numbers are tag counts, except in the “unique genes” columns, which gives the number of unique genes detected, either with the whole library, or with a random sub-sample with 30,000 mapped tags per replicate. Click here for file

Additional file 2: Table S2 Workbook in Excel format containing in its first sheet the full expression data of the R3, L3, L2, L1, and D3 triplicates as counts in CAGE tag clusters, before and after removal of strand-invasion artifacts (signaled by appending “_nw_2” to the sample names), as well as the fold change and corrected p-value for the following pairwise comparisons: L3 vs. R3, R3 vs. D3, and D3 vs. L3. Positive fold changes indicate enrichment in the second member of the comparison, for instance R3 in “L3 vs. R3”. The other sheets of the workbook list the significantly enriched clusters (FDR < 0.1) in one library compared to the other. Click here for file

Additional file 3 **Scripts and programs to reproduce the digital expression comparison between the R3, L3 and D3 libraries.** The HTML file is for display and the Rmd file is its source. Click here for file
