# Supplementary material for: Comparison of RNA- or LNA-hybrid oligonucleotides in template-switching reactions for high-speed sequencing library preparation
Source: BMC Genomics. 2013 Sep 30;14:665. doi: 10.1186/1471-2164-14-665 (PMC3853366; doi:10.1186/1471-2164-14-665)
Supplement: Additional file 2: Table S2 — Workbook in Excel format containing in its first sheet the full expression data of the R3, L3, L2, L1, and D3 triplicates as counts in CAGE tag clusters, before and after removal of strand-invasion artifacts (signaled by appending “_nw_2” to the sample names), as well as the fold change and corrected p-value for the following pairwise comparisons: L3 vs. R3, R3 vs. D3, and D3 vs. L3. Positive fold changes indicate enrichment in the second member of the comparison, for instance R3 in “L3 vs. R3”. The other sheets of the workbook list the significantly enriched clusters (FDR < 0.1) in one library compared to the other. [file 1471-2164-14-665-S2.html]

Comparison of RNA- and LNA-Hybrid Oligonucleotides in Template-Switching Reactions


# Comparison of RNA- and LNA-Hybrid Oligonucleotides in Template-Switching Reactions

This supplementary file documents the commands run to compare the expression
levels of CAGE clusters in libraries made with different template-switching
oligonucleotides. It is also intended as a self-executable tutorial exemplifying
how to make differential gene expression analysis with nanoCAGE in general.

## Table of contents

- Data download and preparation
- Artifact cleaning and alignment of the reads
- Tag clustering
- Annotation
- Preparation for statistical analysis
- Differential representation analysis
  - LNA vs. RNA
  - RNA vs. DNA
  - DNA vs. LNA
- Output of the results as tables
- Notes on the software

## Data download and preparation

The following commands are run in a command-line terminal on Unix systems.

The nanoCAGE library is named `NCms10010`. To better re-use commands, this name is put
in an environment variable.

```
export LIBRARY=NCms10010
```

### Information and download

The `NCms10010` library was made with total RNA from rat muscle. It is
comparing template-switching oligonucleotides that end in RNA ®, DNA (d) or
LNA (l) bases. The comparisons were multiplexed in triplicates.

The data is a single-end MiSeq run (ID: `121012_M00528_0022_AMS2003997-00050`)
of 4,682,200 reads. The output files (`s_G1_L001_R1_001.fastq.gz` and
`s_G1_L001_R1_002.fastq.gz` were decompressed, concatenated, and re-compressed
with `xz`. The resulting file is available for download from the supplementary
material, and from RIKEN. Deposition to DDBJ is in progress.

```
wget http://genome.gsc.riken.jp/plessy-20130430/$LIBRARY.fastq.xz
echo '47b797f36cd20d6548e80e09dc05daa1  NCms10010.fastq.xz' | md5sum -c
```

```
## NCms10010.fastq.xz: OK
```

### De-multiplexing

The barcodes and sample IDs are associated in a whitespace-delimited file
called `NCms10010.id` containing the following. The sample IDs encode the type
of template-switching oligonucleotide used, which each letter representing the
chemical nature of the third, second and first nucleotide, from the 3′ end.

```
cat <<__ID__ > $LIBRARY.id
rrr_1   CACTGA
rrr_2   GCTCTC
rrr_3   TCGCGT
ddd_1   ATCGTG
ddd_2   CACGAT
ddd_3   GTATAC
ddl_1   ACAGAT
ddl_2   CTGACG
ddl_3   GAGTGA
dll_1   AGTAGC
dll_2   GCTGCA
dll_3   TCGAGC
lll_1   ATCATA
lll_2   CGATGA
lll_3   TATAGC
__ID__
```

Samples were demultiplexed with FASTX-toolkit.
The number of extracted reads was collected in a file named `NCms10010.extracted.log`.

```
xzcat $LIBRARY.fastq.xz |
  fastx_barcode_splitter.pl --bcfile $LIBRARY.id --prefix $LIBRARY. --suffix .fq --bol --exact |
  sed 1d | cut -f1,2 |
  perl -ne 'print "extracted\t$_"' |
  grep -v -e unmatched -e total |
  tee $LIBRARY.extracted.log
```

```
## extracted    ddd_1   333255
## extracted    ddd_2   340917
## extracted    ddd_3   148290
## extracted    ddl_1   479148
## extracted    ddl_2   48278
## extracted    ddl_3   37851
## extracted    dll_1   111856
## extracted    dll_2   42939
## extracted    dll_3   55763
## extracted    lll_1   100226
## extracted    lll_2   89915
## extracted    lll_3   61455
## extracted    rrr_1   776221
## extracted    rrr_2   351372
## extracted    rrr_3   186395
```

### Trimming reads to 31 nt

The reads start with 6 bases of barcode, 8 bases of random fingerprint, 4 bases
of spacer, and 3 base of linker, that are all removed in the following command.
The reads were also trimmed in 3′ to ensure that the results were comparable
with a reference HiSeq run (not covered in this document).

```
for FASTQ in *.fq
do
  fastx_trimmer -f 22 -l 52 -Q33 < $FASTQ | sponge $FASTQ
done
```

## Artifact cleaning and alignment of the reads

### Removal of artifacts with TagDust

Download TagDust
and install it in the user's path.

```
cat > tagdust.fa <<__TagDust__
>TS (before barcode)
TAGTCGAACTGAAGGTCTCCAGCA
>RT (without random bases)
TAGTCGAACTGAAGGTCTCCGAACCGCTCTTCCGATCT
>empty (TS linker + RT reverse-complemented)
TATAGGGAGATCGGAAGAGCGGTTCGGAGACCTTCAGTTCGACTA
__TagDust__

for ID in $( awk '{print $1}' $LIBRARY.id )
do
echo -ne "tagdust\t$ID\t"
tagdust tagdust.fa N*.$ID.fq -o $LIBRARY.$ID.dusted.fastq 2>&1 |
  grep -e rejected | cut -f1
done | tee $LIBRARY.tagdust.log
```

```
## tagdust  rrr_1   13723
## tagdust  rrr_2   2625
## tagdust  rrr_3   1596
## tagdust  ddd_1   3691
## tagdust  ddd_2   3927
## tagdust  ddd_3   2432
## tagdust  ddl_1   8646
## tagdust  ddl_2   721
## tagdust  ddl_3   531
## tagdust  dll_1   1786
## tagdust  dll_2   680
## tagdust  dll_3   671
## tagdust  lll_1   1312
## tagdust  lll_2   1293
## tagdust  lll_3   296
```

### Alignment on the rat genome version 4

The following assumes the genome downloaded and indexed for BWA in
the current directory, using `rn4_male` as a base name.

```
GENOME=rn4_male
for FQ in *dusted.fastq
do
  bwa aln -t8 $GENOME -f $(basename $FQ .dusted.fastq).sai $FQ
  bwa samse   $GENOME    $(basename $FQ .dusted.fastq).sai $FQ |
    samtools view -uS - |
    samtools sort - $(basename $FQ .dusted.fastq)
done
```

### Filter reads aligning to rDNA with rRNAdust

`rRNAdust` is available in the supplementary material at http://genome.gsc.riken.jp/plessy-20130430/rRNAdust\_1.02.tar.gz.

Download the reference rRNA sequences at http://www.ncbi.nlm.nih.gov/nuccore/V01270.1 and http://www.ncbi.nlm.nih.gov/gene/170603, and save them in a file called `rat_rDNA.fa`.

```
for ID in $( awk '{print $1}' $LIBRARY.id )
do
  echo -ne "rdna\t$ID\t"
  (rRNAdust -t8 rat_rDNA.fa $LIBRARY.$ID.bam | samtools view -bS  - 2> /dev/null | sponge $LIBRARY.$ID.bam) 2>&1 | sed 's/Excluded: //'
done | tee $LIBRARY.rdna.log
```

```
## rdna rrr_1   239373
## rdna rrr_2   112182
## rdna rrr_3   56075
## rdna ddd_1   56429
## rdna ddd_2   78449
## rdna ddd_3   29489
## rdna ddl_1   178108
## rdna ddl_2   10240
## rdna ddl_3   6743
## rdna dll_1   23612
## rdna dll_2   10327
## rdna dll_3   15254
## rdna lll_1   21777
## rdna lll_2   15454
## rdna lll_3   12189
```

### Alignment statistics

```
for ID in $( awk '{print $1}' $LIBRARY.id )
do
  echo -ne "mapped\t$ID\t"
  (samtools flagstat $LIBRARY.$ID.bam | grep mapped | grep %) | cut -f1 -d' '
done | tee $LIBRARY.mapped.log
```

```
## mapped   rrr_1   469027
## mapped   rrr_2   211307
## mapped   rrr_3   113141
## mapped   ddd_1   259605
## mapped   ddd_2   242952
## mapped   ddd_3   109710
## mapped   ddl_1   265929
## mapped   ddl_2   34369
## mapped   ddl_3   28418
## mapped   dll_1   79426
## mapped   dll_2   27701
## mapped   dll_3   35436
## mapped   lll_1   68930
## mapped   lll_2   65139
## mapped   lll_3   43334
```

### Filter out possible strand-invasion tags

Strand invasion was described in Tang et al, Nucl. Acids Res. (2013) 41
(3):e44, and is more frequent
in LNA- or DNA-based template-switching oligonucleotides.

The following commands need an updated version of the `find_strand_invasion.pl`
that was in Tang et al's supplementary material, where a new `-f` option is
added, with the same semantics as in `samtools`. It is available at
http://genome.gsc.riken.jp/plessy-20130430/find\_strand\_invasion-20130307.pl.

```
ERRORS=2
for BAM in $LIBRARY.???_?.bam
do
  find_strand_invasion.pl -f $BAM -g rn4_male.fa -e $ERRORS -s TATA
done
for ID in $( awk '{print $1}' $LIBRARY.id )
do
  echo -ne "strand-invasion-$ERRORS\t$ID\t"
  (samtools flagstat ${LIBRARY}.${ID}_nw_${ERRORS}_??????_removed_sorted.bam | grep mapped | grep %) | cut -f1 -d' '
done | tee $LIBRARY.strand-invasion-$ERRORS.log
```

```
## strand-invasion-2    rrr_1   51979
## strand-invasion-2    rrr_2   20470
## strand-invasion-2    rrr_3   11137
## strand-invasion-2    ddd_1   201479
## strand-invasion-2    ddd_2   172866
## strand-invasion-2    ddd_3   82123
## strand-invasion-2    ddl_1   130483
## strand-invasion-2    ddl_2   15228
## strand-invasion-2    ddl_3   16489
## strand-invasion-2    dll_1   39596
## strand-invasion-2    dll_2   9538
## strand-invasion-2    dll_3   13259
## strand-invasion-2    lll_1   27204
## strand-invasion-2    lll_2   24179
## strand-invasion-2    lll_3   14127
```

## Tag clustering

Level 1 clusters are single-nucleotide resolution data representing the 5′ ends
of the CAGE tags. Level 2 clusters are groups of level 1 clusters that are
separated by 20 or less nucleotides.

The `level1.py` and `level2.py` scripts implement tag clustering like in the
FANTOM3 and
FANTOM4 projects, and are available at
http://genome.gsc.riken.jp/plessy-20130430/PromoterPipeline\_20130430.tar.gz.

They output their results in Order Switchable Column
(OSC) format, where each line is a
cluster, and each library gives one column counting the tags in the clusters,
and another column where the counts are normalised in parts per million.

See also Carninci et al., Nature Genetics 38
626-635 (2006) and
Suzuki et al., Nature Genetics 41 553-562
(2009) for original
examples of CAGE tag clustering.

```
level1.py -o $LIBRARY.l1.osc.gz -F 516 \
  NCms10010.???_?.bam \
  NCms10010.???_?_nw_?_??????_filtered_sorted.bam

level2.py -o $LIBRARY.l2.osc -t 0 $LIBRARY.l1.osc.gz
gzip $LIBRARY.l2.osc
```

The resulting file NCms10010.l1.osc.gz can be loaded in the
Zenbu system to browse the alignments on
the rat genome.

## Annotation

### Preparation of the reference files.

Data from ENSEMBL 69 were retrieved via
BioMart, with the following XML query.
Note that `external_gene_id` is called *Associated Gene Name* in the web
interface.

```
<?xml version="1.0" encoding="UTF-8"?>
<!DOCTYPE Query>
<Query  virtualSchemaName = "default" formatter = "TSV" header = "0" uniqueRows = "0" count = "" datasetConfigVersion = "0.6" >

    <Dataset name = "rnorvegicus_gene_ensembl" interface = "default" >
        <Attribute name = "ensembl_gene_id" />
        <Attribute name = "ensembl_transcript_id" />
        <Attribute name = "chromosome_name" />
        <Attribute name = "strand" />
        <Attribute name = "external_gene_id" />
        <Attribute name = "transcript_start" />
        <Attribute name = "transcript_end" />
        <Attribute name = "gene_biotype" />
        <Attribute name = "exon_chrom_start" />
        <Attribute name = "exon_chrom_end" />
    </Dataset>
</Query>
```

Convert coordinates to gene names

```
cat mart_export.txt | 
  sed -e 1d -e 's|\t\t|\tno_symbol\t|'           |
  awk '{OFS="\t"} {print $3, $6, $7, $5, 0, $4}' |
  grep -v ^[JA]                                  |
  uniq                                           |
  sed -e 's/-1$/-/'                              \
      -e 's/1$/+/'                               \
      -e 's/^/chr/'                              |
  sort -k1,1 -k2,2n                              \
  > rn4_male.symbols.bed


### Gene symbols
```

```
zcat $LIBRARY.l1.osc.gz | grep -v \# | sed 1d | awk '{OFS="\t"}{print $2, $3, $4, "l2", "1000", $5}' > $LIBRARY.l1.bed
zcat $LIBRARY.l2.osc.gz | grep -v \# | sed 1d | awk '{OFS="\t"}{print $2, $3, $4, "l2", "1000", $5}' > $LIBRARY.l2.bed
zcat $LIBRARY.l2.osc.gz | grep -v \# | sed 1d | awk '{OFS="\t"}{print $2, $3, $4, "l2", "1000", $5}' > $LIBRARY.l2.bed

bedtools intersect -a $LIBRARY.l2.bed -b rn4_male.symbols.bed -s -loj |
  awk '{OFS="\t"}{print $1":"$2"-"$3$6,$10}' | 
  bedtools groupby -g 1 -c 2 -o distinct > $LIBRARY.l2.genes
```

### Repeated elements

Download the repeatmasker track from the UCSC genome browser and save it in a file called `rn4_male.repeatmasker.bed`.

```
bedtools intersect -a $LIBRARY.l2.bed -b rn4_male.repeatmasker.bed -s -loj |
  awk '{OFS="\t"}{print $1":"$2"-"$3$6,$10}' |
  bedtools groupby -g 1 -c 2 -o distinct > $LIBRARY.l2.rmsk
```

## Preparation for statistical analysis

The following commands are run in the `R` package for statistical computing.

### Load the data.

The oscR library is available at https://github.com/charles-plessy/oscR.

The following commands load the level 1 and 2 clusters into data frames where
the column names correspond to the sample IDs defined above.

```
LIBRARY <- system("echo $LIBRARY", intern = TRUE)

library(oscR)

l1 <- read.osc(paste(LIBRARY, "l1", "osc", "gz", sep = "."), drop.coord = T, 
    drop.norm = T)
l2 <- read.osc(paste(LIBRARY, "l2", "osc", "gz", sep = "."), drop.coord = T, 
    drop.norm = T)

colnames(l1) <- sub("raw.NCms1.....", "", colnames(l1))
colnames(l2) <- sub("raw.NCms1.....", "", colnames(l2))

colnames(l1) <- sub("_......_filtered_sorted", "", colnames(l1))
colnames(l2) <- sub("_......_filtered_sorted", "", colnames(l2))
```

### Organise the data.

The following commands defined convenient shortcuts to manipulates groups of
libraries. The presence of `nw_2` in the names indicate that strand-invasion
artifacts have been removed.

```
ddd <- c("ddd_1", "ddd_2", "ddd_3")
ddl <- c("ddl_1", "ddl_2", "ddl_3")
dll <- c("dll_1", "dll_2", "dll_3")
lll <- c("lll_1", "lll_2", "lll_3")
rrr <- c("rrr_1", "rrr_2", "rrr_3")
all <- c(rrr, lll, dll, ddl, ddd)

ddd_nw_2 <- c("ddd_1_nw_2", "ddd_2_nw_2", "ddd_3_nw_2")
ddl_nw_2 <- c("ddl_1_nw_2", "ddl_2_nw_2", "ddl_3_nw_2")
dll_nw_2 <- c("dll_1_nw_2", "dll_2_nw_2", "dll_3_nw_2")
lll_nw_2 <- c("lll_1_nw_2", "lll_2_nw_2", "lll_3_nw_2")
rrr_nw_2 <- c("rrr_1_nw_2", "rrr_2_nw_2", "rrr_3_nw_2")
all_nw_2 <- c(rrr_nw_2, lll_nw_2, dll_nw_2, ddl_nw_2, ddd_nw_2)
```

```
TPM <- function(clusters) {
    clusters.tpm <- data.frame(prop.table(as.matrix(clusters), 2) * 1e+06)
    colnames(clusters.tpm) <- colnames(clusters)
    return(clusters.tpm)
}

L2 <- TPM(l2)

L2.means <- data.frame(ddd = apply(L2[, ddd], 1, mean), ddl = apply(L2[, ddl], 
    1, mean), dll = apply(L2[, dll], 1, mean), lll = apply(L2[, lll], 1, mean), 
    rrr = apply(L2[, rrr], 1, mean))

L2.means_nw_2 <- data.frame(ddd = apply(L2[, ddd_nw_2], 1, mean), ddl = apply(L2[, 
    ddl_nw_2], 1, mean), dll = apply(L2[, dll_nw_2], 1, mean), lll = apply(L2[, 
    lll_nw_2], 1, mean), rrr = apply(L2[, rrr_nw_2], 1, mean))

L2.sd <- data.frame(ddd = apply(L2[, ddd], 1, sd), ddl = apply(L2[, ddl], 1, 
    sd), dll = apply(L2[, dll], 1, sd), lll = apply(L2[, lll], 1, sd), rrr = apply(L2[, 
    rrr], 1, sd))
```

### Annotation of the results

```
genesymbols <- read.table(paste(LIBRARY, "l2", "genes", sep = "."), col.names = c("cluster", 
    "symbol"))
rownames(genesymbols) <- genesymbols$cluster
genesymbols$rmsk <- read.table(paste(LIBRARY, "l2", "rmsk", sep = "."), col.names = c("cluster", 
    "rmsk"))[, "rmsk"]
```

## Differential representation analysis

Statistical comparisons using edgeR.

```
library(edgeR)
```

The following plots represent:

- the multidimensional scaling of the samples,
  where spatial separation between the two sets of triplicates indicates that the
  factor that is compared (type of template-switching oligonucleotide) introduces
  more differences that the simple technical fluctuations,
- the expression levels of the CAGE clusters as a M-A plot, where dots in red are clusters
  significantly enriched in one type of libraries. Vertical distance from the
  horizontal midline represent the amplitude of the differences, and distance on
  the horizontal axis represents the average strength of expression.

The following comparisons show the difference (or lack of it) between
non-filtered and filtered data, and then explore the filtered data in more
details.

### LNA vs. RNA

```
x <- DGEList(counts = l2[, c(lll, rrr)], group = c(rep("lll", 3), rep("rrr", 
    3)), remove.zeros = TRUE)
```

```
## Removing 114680 rows with all zero counts.
```

```
x <- calcNormFactors(x)
x <- estimateCommonDisp(x)
x <- estimateTagwiseDisp(x)
x.com <- exactTest(x)
lr <- x
lr.com <- x.com
plotMDS(lr)
```

```
plotSmear(lr.com, de.tags = rownames(lr.com)[decideTestsDGE(lr.com) != 0], cex = 0.8, 
    main = "LNA / RNA", ylab = "LNA (bottom) / RNA (top)")
```

```
x <- DGEList(counts = l2[, c(lll_nw_2, rrr_nw_2)], group = c(rep("lll_nw_2", 
    3), rep("rrr_nw_2", 3)), remove.zeros = TRUE)
```

```
## Removing 123000 rows with all zero counts.
```

```
x <- calcNormFactors(x)
x <- estimateCommonDisp(x)
x <- estimateTagwiseDisp(x)
x.com <- exactTest(x)
lr_nw_2 <- x
lr_nw_2.com <- x.com
plotMDS(lr_nw_2)
```

```
plotSmear(lr_nw_2.com, de.tags = rownames(lr_nw_2.com)[decideTestsDGE(lr_nw_2.com) != 
    0], cex = 0.8, main = "LNA / RNA (filtered)", ylab = "LNA (bottom) / RNA (top)")
```

```
lr_nw_2.up <- sum(decideTestsDGE(lr_nw_2.com) > 0)
lr_nw_2.down <- sum(decideTestsDGE(lr_nw_2.com) < 0)
```

866 clusters were enriched and 44 were depleted in RNA
libraries compared to LNA. The top 100 stronger fold changes in each direction are shown below.

```
# Summary of the top 100 clusters enriched in RNA libraries.
summary(merge(subset(topTags(lr_nw_2.com, Inf)$table, logFC > 0)[1:100, ], genesymbols[, 
    -1], by = 0, sort = FALSE))
```

```
##   Row.names             logFC           logCPM          PValue              FDR               symbol        rmsk   
##  Length:100         Min.   : 2.03   Min.   : 7.27   Min.   :0.00e+00   Min.   :0.00e+00   .      :40   .      :86  
##  Class :AsIs        1st Qu.: 4.93   1st Qu.: 8.07   1st Qu.:0.00e+00   1st Qu.:0.00e+00   Myh2   : 3   7SLRNA :11  
##  Mode  :character   Median : 6.55   Median : 8.86   Median :0.00e+00   Median :0.00e+00   Eno3   : 2   GC_rich: 1  
##                     Mean   : 6.45   Mean   : 9.69   Mean   :1.03e-15   Mean   :1.47e-12   Myl1   : 2   L1MCa  : 1  
##                     3rd Qu.: 7.96   3rd Qu.:11.12   3rd Qu.:1.24e-16   3rd Qu.:2.22e-13   Acat1  : 1   L1_Rat1: 1  
##                     Max.   :11.82   Max.   :16.94   Max.   :1.11e-14   Max.   :1.50e-11   Aco2   : 1   4.5SRNA: 0  
##                                                                                           (Other):51   (Other): 0
```

```
# Top 15 clusters enriched in RNA libraries.
merge(subset(topTags(lr_nw_2.com, Inf)$table, logFC > 0)[1:15, ], genesymbols[, 
    -1], by = 0, sort = FALSE)
```

```
##                    Row.names  logFC logCPM     PValue        FDR symbol   rmsk
## 1      chr5:5309920-5309978-  9.729 11.305 4.054e-109 5.622e-104      . 7SLRNA
## 2    chr8:62243583-62243658+  8.946 11.343  2.517e-87  1.745e-82      . 7SLRNA
## 3    chr9:65727648-65727774-  6.269 12.740  7.765e-76  3.556e-71   Myl1      .
## 4   chr11:68375411-68375528-  7.333 11.185  1.026e-75  3.556e-71      . 7SLRNA
## 5   chr12:22851453-22851493-  7.585 11.187  1.724e-64  4.781e-60      . 7SLRNA
## 6    chr3:70682541-70682599+  9.607 11.185  6.080e-61  1.405e-56      . 7SLRNA
## 7    chr6:91117279-91117393+  8.852 11.247  1.312e-60  2.599e-56      . 7SLRNA
## 8    chrX:54985894-54985915+ 11.819 11.312  3.036e-55  5.262e-51      . 7SLRNA
## 9  chr2:145713673-145713772+  2.927 14.250  1.063e-53  1.637e-49      .      .
## 10         chrM:12334-13779+  2.595 12.261  1.519e-53  2.106e-49      .      .
## 11 chr1:202745119-202745217+  6.467 14.539  1.020e-51  1.286e-47      .      .
## 12   chr7:68333299-68333423-  8.331 11.253  3.925e-51  4.535e-47      . 7SLRNA
## 13   chr2:77482169-77482291+  8.263 11.194  2.203e-48  2.350e-44 Znf622 7SLRNA
## 14  chr10:59473911-59473930+  7.875 11.178  2.466e-47  2.443e-43      . 7SLRNA
## 15   chr1:85599001-85599082+  6.291  8.801  1.865e-39  1.724e-35  Hspb6      .
```

```
# Summary of the top 100 clusters enriched in LNA libraries.
summary(merge(subset(topTags(lr_nw_2.com, Inf)$table, logFC < 0)[1:100, ], genesymbols[, 
    -1], by = 0, sort = FALSE))
```

```
##   Row.names             logFC           logCPM          PValue              FDR             symbol                  rmsk   
##  Length:100         Min.   :-7.96   Min.   : 5.37   Min.   :0.000000   Min.   :0.0000   .      :66   .                :50  
##  Class :AsIs        1st Qu.:-5.46   1st Qu.: 5.72   1st Qu.:0.000056   1st Qu.:0.0108   Armc2  : 3   (CAGAGA)n        :31  
##  Mode  :character   Median :-4.46   Median : 6.12   Median :0.000420   Median :0.0609   Nrd1   : 2   GA-rich          : 5  
##                     Mean   :-4.41   Mean   : 6.70   Mean   :0.000874   Mean   :0.1001   Sesn1  : 2   (AGTAG)n,(TAGGG)n: 1  
##                     3rd Qu.:-3.50   3rd Qu.: 7.15   3rd Qu.:0.001418   3rd Qu.:0.1664   Acbd3  : 1   B1_Mur3          : 1  
##                     Max.   :-1.20   Max.   :12.55   Max.   :0.003802   Max.   :0.3682   Chordc1: 1   B1_Rn            : 1  
##                                                                                         (Other):25   (Other)          :11
```

Positive fold change indicate enrichment in RNA libraries. The 7SLRNA hits
are concentrated at the top of the list. LNA libraries are enriched for hits
on CAGAGA repeats, even beyond the significance level (FDR) of the statistical
comparison.

```
library(reshape)
```

```
## Loading required package: plyr
```

```
## Attaching package: 'reshape'
```

```
## The following object is masked from 'package:plyr':
## 
## rename, round_any
```

```
library(ggplot2)
srprna <- rownames(subset(genesymbols, rmsk == "7SLRNA"))
srprna.expression <- melt(L2[srprna, c(rrr_nw_2, ddd_nw_2, lll_nw_2)], measure.vars = c(rrr_nw_2, ddd_nw_2, lll_nw_2))
for (group in c("ddd", "lll", "rrr")) srprna.expression[grep(group, srprna.expression$variable), "group"] <- group
srprna.expression$group <- reorder(factor(srprna.expression$group), srprna.expression$value, function(x) sum(x) * -1)
qplot(data = srprna.expression, value, reorder(variable, value, sum), xlab = "Parts per million", ylab = "Library", main = "Expression levels of 7SL RNA genes", 
    col = group)
```

The measured expression of 7SL RNA is strongest in RNA libraries, strong in DNA
libraries, and weak in LNA libraries.

### RNA vs. DNA

```
x <- DGEList(counts = l2[, c(rrr, ddd)], group = factor(c(rep("rrr", 3), rep("ddd", 3)), levels = c("rrr", "ddd")), remove.zeros = TRUE)
```

```
## Removing 89641 rows with all zero counts.
```

```
x <- calcNormFactors(x)
x <- estimateCommonDisp(x)
x <- estimateTagwiseDisp(x)
x.com <- exactTest(x)
rd <- x
rd.com <- x.com
plotMDS(x)
```

```
plotSmear(rd.com, de.tags = rownames(rd.com)[decideTestsDGE(rd.com) != 0], cex = 0.8, main = "RNA / DNA", ylab = "RNA (bottom) / LNA (top)")
```

```
x <- DGEList(counts = l2[, c(rrr_nw_2, ddd_nw_2)], group = factor(c(rep("rrr_nw_2", 3), rep("ddd_nw_2", 3)), levels = c("rrr_nw_2", "ddd_nw_2")), remove.zeros = TRUE)
```

```
## Removing 106919 rows with all zero counts.
```

```
x <- calcNormFactors(x)
x <- estimateCommonDisp(x)
x <- estimateTagwiseDisp(x)
x.com <- exactTest(x)
plotMDS(x)
```

```
rd_nw_2 <- x
rd_nw_2.com <- x.com
plotSmear(rd_nw_2.com, de.tags = rownames(rd_nw_2.com)[decideTestsDGE(rd_nw_2.com) != 0], cex = 0.8, main = "RNA / DNA (filtered)", ylab = "RNA (bottom) / DNA (top)")
```

626 clusters were enriched and 87 were depleted in RNA
libraries compared to DNA.

The majority of the clusters in the top 100 enriched in the RNA libraries did
not overlap with repeat elements, and were overlapping with loci having gene
symbols. In contrast, the majority of the top 100 clusters enriched in the DNA
libraries did not overlap with known genes. A mild enrichment for GGGTG
simple repeats is noted.

```
# Summary of the top 100 clusters enriched in DNA libraries.
summary(merge(subset(topTags(rd_nw_2.com, Inf)$table, logFC > 0)[1:100, ], genesymbols[, -1], by = 0, sort = FALSE))
```

```
##   Row.names             logFC           logCPM          PValue              FDR                   symbol                rmsk   
##  Length:100         Min.   : 1.41   Min.   : 5.21   Min.   :0.00e+00   Min.   :0.00000   .           :85   .              :81  
##  Class :AsIs        1st Qu.: 4.70   1st Qu.: 5.60   1st Qu.:0.00e+00   1st Qu.:0.00001   F1LY53_RAT  : 5   (GGGTG)n       : 4  
##  Mode  :character   Median : 5.76   Median : 6.23   Median :3.70e-06   Median :0.00145   F1M1V3_RAT  : 2   GA-rich        : 3  
##                     Mean   : 5.84   Mean   : 6.84   Mean   :8.09e-05   Mean   :0.01749   D3ZWL6_RAT  : 1   G-rich         : 3  
##                     3rd Qu.: 6.81   3rd Qu.: 7.63   3rd Qu.:1.07e-04   3rd Qu.:0.02649   Kpnb1       : 1   (CAGA)n,GA-rich: 2  
##                     Max.   :10.54   Max.   :13.73   Max.   :5.20e-04   Max.   :0.09911   LOC100364794: 1   (CAGA)n        : 1  
##                                                                                          (Other)     : 5   (Other)        : 6
```

```
# Summary of the top 100 clusters enriched in RNA libraries.
summary(merge(subset(topTags(rd_nw_2.com, Inf)$table, logFC < 0)[1:100, ], genesymbols[, -1], by = 0, sort = FALSE))
```

```
##   Row.names             logFC           logCPM          PValue              FDR               symbol        rmsk   
##  Length:100         Min.   :-9.17   Min.   : 6.92   Min.   :0.00e+00   Min.   :0.00e+00   .      :36   .      :91  
##  Class :AsIs        1st Qu.:-6.09   1st Qu.: 7.62   1st Qu.:0.00e+00   1st Qu.:0.00e+00   7SK    : 1   7SLRNA : 6  
##  Mode  :character   Median :-5.03   Median : 8.06   Median :2.00e-15   Median :4.00e-12   Acat1  : 1   7SK    : 1  
##                     Mean   :-5.15   Mean   : 8.79   Mean   :3.38e-13   Mean   :5.07e-10   Aco2   : 1   GC_rich: 1  
##                     3rd Qu.:-3.93   3rd Qu.: 9.17   3rd Qu.:7.00e-14   3rd Qu.:1.30e-10   Actb   : 1   L1MCa  : 1  
##                     Max.   :-1.83   Max.   :16.75   Max.   :3.99e-12   Max.   :5.61e-09   Atp5j  : 1   4.5SRNA: 0  
##                                                                                           (Other):59   (Other): 0
```

### DNA vs. LNA

```
x <- DGEList(counts = l2[, c(ddd, lll)], group = c(rep("ddd", 3), rep("lll", 3)), remove.zeros = TRUE)
```

```
## Removing 168500 rows with all zero counts.
```

```
x <- calcNormFactors(x)
x <- estimateCommonDisp(x)
x <- estimateTagwiseDisp(x)
x.com <- exactTest(x)
dl <- x
dl.com <- x.com
plotMDS(x)
```

```
plotSmear(dl.com, de.tags = rownames(dl.com)[decideTestsDGE(dl.com) != 0], cex = 0.8, main = "DNA / LNA", ylab = "DNA (bottom) / LNA (top)")
```

```
x <- DGEList(counts = l2[, c(ddd_nw_2, lll_nw_2)], group = c(rep("ddd_nw_2", 3), rep("lll_nw_2", 3)), remove.zeros = TRUE)
```

```
## Removing 190706 rows with all zero counts.
```

```
x <- calcNormFactors(x)
x <- estimateCommonDisp(x)
x <- estimateTagwiseDisp(x)
x.com <- exactTest(x)
plotMDS(x)
```

```
dl_nw_2 <- x
dl_nw_2.com <- x.com
plotSmear(dl_nw_2.com, de.tags = rownames(dl_nw_2.com)[decideTestsDGE(dl_nw_2.com) != 0], cex = 0.8, main = "DNA / LNA (filtered)", ylab = "DNA (bottom) / LNA (top)")
```

5 clusters were enriched and 39 were depleted in LNA
libraries compared to DNA.

After filtering out strand-invasion artifacts, only few significant differences
remain between the DNA and LNA libraries. 7SLRNA was also depleted in LNA
libraries, and CAGAGA repeats were enriched.

```
summary(decideTestsDGE(dl_nw_2.com))
```

```
##    [,1] 
## -1    39
## 0  70908
## 1      5
```

```
summary(merge(subset(topTags(dl_nw_2.com, Inf)$table, logFC > 0)[1:100, ], genesymbols[, -1], by = 0, sort = FALSE))
```

```
##   Row.names             logFC           logCPM          PValue             FDR               symbol                  rmsk   
##  Length:100         Min.   :0.896   Min.   : 6.36   Min.   :0.00000   Min.   :0.000   .         :49   .                :62  
##  Class :AsIs        1st Qu.:2.921   1st Qu.: 6.76   1st Qu.:0.00239   1st Qu.:1.000   D4A4B0_RAT: 3   (CAGAGA)n        :21  
##  Mode  :character   Median :3.923   Median : 7.36   Median :0.00875   Median :1.000   Mbnl2     : 2   GA-rich          : 5  
##                     Mean   :3.922   Mean   : 7.85   Mean   :0.00976   Mean   :0.871   NEXN_RAT  : 2   B1_Mur3          : 2  
##                     3rd Qu.:4.866   3rd Qu.: 8.26   3rd Qu.:0.01569   3rd Qu.:1.000   Nrd1      : 2   (AGTAG)n,(TAGGG)n: 1  
##                     Max.   :9.389   Max.   :13.91   Max.   :0.02512   Max.   :1.000   Nsbp1     : 2   B4A              : 1  
##                                                                                       (Other)   :40   (Other)          : 8
```

```
summary(merge(subset(topTags(dl_nw_2.com, Inf)$table, logFC < 0)[1:100, ], genesymbols[, -1], by = 0, sort = FALSE))
```

```
##   Row.names             logFC            logCPM          PValue              FDR                symbol                rmsk   
##  Length:100         Min.   :-8.447   Min.   : 6.71   Min.   :0.00e+00   Min.   :0.0000   .         :57   .              :79  
##  Class :AsIs        1st Qu.:-5.815   1st Qu.: 7.60   1st Qu.:3.40e-06   1st Qu.:0.0085   Myh2      :11   7SLRNA         :11  
##  Mode  :character   Median :-4.765   Median : 8.68   Median :1.54e-04   Median :0.1882   F1LY53_RAT: 3   (GGGTG)n       : 3  
##                     Mean   :-4.647   Mean   : 8.68   Mean   :5.67e-04   Mean   :0.3550   Col3a1    : 2   G-rich         : 2  
##                     3rd Qu.:-3.204   3rd Qu.: 9.19   3rd Qu.:8.75e-04   3rd Qu.:0.6733   Myl1      : 2   (CAGA)n        : 1  
##                     Max.   :-0.776   Max.   :15.64   Max.   :2.46e-03   Max.   :1.0000   Tnnt3     : 2   (CAGA)n,GA-rich: 1  
##                                                                                          (Other)   :23   (Other)        : 3
```

```
save.image("analysis.RData")
```

## Output of the results as tables

```
# One table per list of significantly over-represented clusters.
write.csv(file = "R-L.csv", merge(subset(topTags(lr_nw_2.com, Inf)$table, logFC > 0 & FDR < 0.1), genesymbols[, -1], by = "row.names", all.x = "T", sort = FALSE), 
    row.names = FALSE)
write.csv(file = "L-R.csv", merge(subset(topTags(lr_nw_2.com, Inf)$table, logFC < 0 & FDR < 0.1), genesymbols[, -1], by = "row.names", all.x = "T", sort = FALSE), 
    row.names = FALSE)
write.csv(file = "D-R.csv", merge(subset(topTags(rd_nw_2.com, Inf)$table, logFC > 0 & FDR < 0.1), genesymbols[, -1], by = "row.names", all.x = "T", sort = FALSE), 
    row.names = FALSE)
write.csv(file = "R-D.csv", merge(subset(topTags(rd_nw_2.com, Inf)$table, logFC < 0 & FDR < 0.1), genesymbols[, -1], by = "row.names", all.x = "T", sort = FALSE), 
    row.names = FALSE)
write.csv(file = "L-D.csv", merge(subset(topTags(dl_nw_2.com, Inf)$table, logFC > 0 & FDR < 0.1), genesymbols[, -1], by = "row.names", all.x = "T", sort = FALSE), 
    row.names = FALSE)
write.csv(file = "D-L.csv", merge(subset(topTags(dl_nw_2.com, Inf)$table, logFC < 0 & FDR < 0.1), genesymbols[, -1], by = "row.names", all.x = "T", sort = FALSE), 
    row.names = FALSE)

# One summary table combining all the results.
l2[rownames(topTags(lr_nw_2.com, Inf)), "LR.logFC"] <- topTags(lr_nw_2.com, Inf)$table$logFC
l2[rownames(topTags(lr_nw_2.com, Inf)), "LR.FDR"] <- topTags(lr_nw_2.com, Inf)$table$FDR
l2[rownames(topTags(rd_nw_2.com, Inf)), "RD.logFC"] <- topTags(rd_nw_2.com, Inf)$table$logFC
l2[rownames(topTags(rd_nw_2.com, Inf)), "RD.FDR"] <- topTags(rd_nw_2.com, Inf)$table$FDR
l2[rownames(topTags(dl_nw_2.com, Inf)), "DL.logFC"] <- topTags(dl_nw_2.com, Inf)$table$logFC
l2[rownames(topTags(dl_nw_2.com, Inf)), "DL.FDR"] <- topTags(dl_nw_2.com, Inf)$table$FDR
l2$symbol <- genesymbols$symbol
l2$rmsk <- genesymbols$rmsk
write.csv(file = paste(LIBRARY, "DGE", "csv", sep = "."), l2)
```

## Notes on the software

This analysis was done on a iMac with a i7 hyperthreaded quad-core CPU (2.93
GHz) and 12 GiB of memory, running Debian system, with
the following packages installed.

- bedtools 2.17.0-1
- bwa 0.6.2-2
- fastx-toolkit 0.0.13.2-1
- moreutils 0.47 (for the `sponge` command)
- r-base (see below)
- r-bioc-edgeR (see below)
- samtools 0.1.19-1

This tutorial was made with the knitr library for
`R`, that produces HTML pages from templates containing executable code.

```
sessionInfo()
```

```
## R version 3.0.1 (2013-05-16)
## Platform: x86_64-pc-linux-gnu (64-bit)
## 
## locale:
##  [1] LC_CTYPE=en_GB.UTF-8       LC_NUMERIC=C               LC_TIME=en_GB.UTF-8        LC_COLLATE=en_GB.UTF-8     LC_MONETARY=en_GB.UTF-8   
##  [6] LC_MESSAGES=en_GB.UTF-8    LC_PAPER=C                 LC_NAME=C                  LC_ADDRESS=C               LC_TELEPHONE=C            
## [11] LC_MEASUREMENT=en_GB.UTF-8 LC_IDENTIFICATION=C       
## 
## attached base packages:
## [1] methods   stats     graphics  grDevices utils     datasets  base     
## 
## other attached packages:
## [1] ggplot2_0.9.3.1 reshape_0.8.4   plyr_1.8        edgeR_3.2.4     limma_3.16.7    oscR_0.1.1     
## 
## loaded via a namespace (and not attached):
##  [1] codetools_0.2-8    colorspace_1.2-2   dichromat_2.0-0    digest_0.6.3       evaluate_0.4.3     formatR_0.7        grid_3.0.1        
##  [8] gtable_0.1.2       knitr_1.2          labeling_0.1       MASS_7.3-26        munsell_0.4        proto_0.3-10       RColorBrewer_1.0-5
## [15] reshape2_1.2.2     scales_0.2.3       stringr_0.6.2      tools_3.0.1
```
